# Bronchodilator responsiveness and future chronic airflow obstruction: a multinational longitudinal study

**DOI:** 10.1016/j.eclinm.2025.103123

**Published:** 2025-02-21

**Authors:** Ben Knox-Brown, Fahad Algharbi, Octavia Mulhern, James Potts, Imed Harrabi, Christer Janson, Rune Nielsen, Dhiraj Agarwal, Andrei Malinovschi, Sanjay Juvekar, Miriam Denguezli, Thorarinn Gíslason, Rana Ahmed, Asaad Nafees, Parvaiz A. Koul, Daniel Obaseki, Mahesh Padukudru Anand, Li Cher Loh, Hermínia Brites Dias, Fátima Rodrigues, David Mannino, Mohammed Elbiaze, Karima El Rhazi, Filip Mejza, Graham Devereux, Frits M.E. Franssen, Asma El Sony, Emiel Wouters, Mohammed Al Ghobain, Kevin Mortimer, Abdul Rashid, Rashid Osman, Michael Studnicka, Joao Cardoso, Peter Burney, Andre F.S. Amaral, BOLD Collaborative Research Groupao, Hasan Hafizi, Anila Aliko, Donika Bardhi, Holta Tafa, Natasha Thanasi, Arian Mezini, Alma Teferici, Dafina Todri, Jolanda Nikolla, Rezarta Kazasi, Hamid Hacene Cherkaski, Amira Bengrait, Tabarek Haddad, Ibtissem Zgaoula, Maamar Ghit, Abdelhamid Roubhia, Soumaya Boudra, Feryal Atoui, Randa Yakoubi, Rachid Benali, Abdelghani Bencheikh, Nadia Ait-Khaled, Christine Jenkins, Guy Marks, Tessa Bird, Paola Espinel, Kate Hardaker, Brett Toelle, Michael Studnicka, Torkil Dawes, Bernd Lamprecht, Lea Schirhofer, Herve Lawin, Arsene Kpangon, Karl Kpossou, Gildas Agodokpessi, Paul Ayelo, Benjamin Fayomi, Rolus Atrokpo, Gaston Hounton, Dieudonnè Yadjodo, Bertrand Mbatchou, Atongno Humphrey Ashu, Wan C. Tan, Wen Wang, NanShan Zhong, Shengming Liu, Jiachun Lu, Pixin Ran, Dali Wang, Jin-ping Zheng, Yumin Zhou, Rain Jõgi, Hendrik Laja, Katrin Ulst, Vappu Zobel, Toomas-Julius Lill, Katrin Kiili, Ira Laanelepp, Tobias Welte, Isabelle Bodemann, Henning Geldmacher, Alexandra Schweda-Linow, Thorarinn Gislason, Bryndis Benedikdtsdottir, Kristin Jörundsdottir, Lovisa Gudmundsdottir, Sigrun Gudmundsdottir, Gunnar Gudmundsson, Elin Helga Thorarinsdottir, Hjördis Sigrun Pálsdottir, Mahesh Padukudru Anand, Parvaiz A. Koul, Sajjad Malik, Nissar A. Hakim, Umar Hafiz Khan, Rohini Chowgule, Vasant Shetye, Jonelle Raphael, Rosel Almeda, Mahesh Tawde, Rafiq Tadvi, Sunil Katkar, Milind Kadam, Rupesh Dhanawade, Umesh Ghurup, Sanjay Juvekar, Siddhi Hirve, Somnath Sambhudas, Bharat Chaidhary, Meera Tambe, Savita Pingale, Arati Umap, Archana Umap, Nitin Shelar, Sampada Devchakke, Sharda Chaudhary, Suvarna Bondre, Savita Walke, Ashleshsa Gawhane, Anil Sapkal, Rupali Argade, Vijay Gaikwad, Dhiraj Agrawal, Babu Pawar, Shalan Mhetre, Namdev Kale, Shirish Kathale, Sundeep Salvi, Bill Brashier, Jyoti Londhe, Sapna Madas, Althea Aquart-Stewart, Akosua Francia Aikman, Talant M. Sooronbaev, Bermet M. Estebesova, Meerim Akmatalieva, Saadat Usenbaeva, Jypara Kydyrova, Eliza Bostonova, Ulan Sheraliev, Nuridin Marajapov, Nurgul Toktogulova, Berik Emilov, Toktogul Azilova, Gulnara Beishekeeva, Nasyikat Dononbaeva, AijamalTabyshova, Kevin Mortimer, Wezzie Nyapigoti, Ernest Mwangoka, Mayamiko Kambwili, Martha Chipeta, Gloria Banda, Suzgo Mkandawire, Justice Banda, Graham Devereux, Jamie Rylance, Martin Njoroge, Catherine Chirwa, Chifundo Mhango, Edgar Ngwira, Faith Zumazuma, Frank Jonas, Patrick Mjojo, Li-Cher Loh, Abdul Rashid, Siti Sholehah, Mohamed C. Benjelloun, Chakib Nejjari, Mohamed Elbiaze, Karima El Rhazi, Manelle Rjimati, Btissame ElHarche, Reda Benjelloun, Yassin Chefchaou, E.F.M. Wouters, G.J. Wesseling, Daniel Obaseki, Gregory Erhabor, Olayemi Awopeju, Olufemi Adewole, Amund Gulsvik, Tina Endresen, Lene Svendsen, Rune Nielsen, Marit Aardal, Hildegunn B. Fleten, Gerd Eli Dale, Eli Nordeide, Malin P. Grøttveit, Åsa Skjelde, Ane Aamli Gagnat, Anders Ørskov Rotevatn, Marta Erdal, Asaad A. Nafees, Muhammad Irfan, Hasan Nawaz Tahir, Muhammad Noman, Roman Ul Haq, Luisito F. Idolor, Teresita S. de Guia, Norberto A. Francisco, Camilo C. Roa, Fernando G. Ayuyao, Cecil Z. Tady, Daniel T. Tan, Sylvia Banal-Yang, Vincent M. Balanag, Maria Teresita N Reyes, Renato B. Dantes, Stefanni Nonna M Paraguas, Renato B. Dantes, Lourdes Amarillo, Lakan U. Berratio, Lenora C. Fernandez, Norberto A. Francisco, Gerard S. Garcia, Teresita S. de Guia, Luisito F. Idolor, Sullian S. Naval, Thessa Reyes, Camilo C. Roa, Ma Flordeliza Sanchez, Leander P. Simpao, Ewa Nizankowska-Mogilnicka, Jakub Frey, Rafal Harat, Filip Mejza, Pawel Nastalek, Andrzej Pajak, Wojciech Skucha, Andrzej Szczeklik, Magda Twardowska, Cristina Bárbara, Fátima Rodrigues, Hermínia Dias, João Cardoso, João Almeida, Maria João Matos, Paula Simão, Moutinho Santos, Reis Ferreira, M. Al Ghobain, H. Alorainy, E. El-Hamad, M. Al Hajjaj, A. Hashi, R. Dela, R. Fanuncio, E. Doloriel, I. Marciano, L. Safia, Eric Bateman, Anamika Jithoo, Desiree Adams, Edward Barnes, Jasper Freeman, Anton Hayes, Sipho Hlengwa, Christine Johannisen, Mariana Koopman, Innocentia Louw, Ina Ludick, Alta Olckers, Johanna Ryck, Janita Storbeck, Richard van Zyl-Smit, Kirthi Gunasekera, Rajitha Wickremasinghe, Asma Elsony, Hana A. Elsadig, Nada Bakery Osman, Bandar Salah Noory, Monjda Awad Mohamed, Hasab Alrasoul Akasha Ahmed Osman, Namarig Moham ed Elhassan, Abdel Mu‘is El Zain, Marwa Mohamed Mohamaden, Suhaiba Khalifa, Mahmoud Elhadi, Mohand Hassan, Dalia Abdelmonam, Rana Ahmed, Rashid Osman, Hind Eltigani, Najlaa Mohamed Abass, Ahmed Beriar Ahmed, Sahar AlaElddin, Christer Janson, Inga Sif Olafsdottir, Katarina Nisser, Ulrike Spetz-Nyström, Gunilla Hägg, Gun-Marie Lund, Andrei Malinovschi, Eva Wallberg, Birgitta Appelfeldt, Mona Andrén, Terence Seemungal, Fallon Lutchmansingh, Liane Conyette, Imed Harrabi, Myriam Denguezli, Zouhair Tabka, Hager Daldoul, Zaki Boukheroufa, Firas Chouikha, Wahbi Belhaj Khalifa, Safa Hsan, Nadia Lakhdar, Mounir Landolsi, Ali Kocabaş, Attila Hancioglu, Ismail Hanta, Sedat Kuleci, Ahmet Sinan Turkyilmaz, Sema Umut, Turgay Unalan, Peter G.J. Burney, Anamika Jithoo, Louisa Gnatiuc, Hadia Azar, Jaymini Patel, Caron Amor, James Potts, Michael Tumilty, Fiona McLean, Risha Dudhaiya, Andre F.S. Amaral, Octavia Mulhern, Emmanouil Bagkeris, Jasleen Gegic, Paul Cullinan, Cosetta Minelli, A Sonia Buist, Mary Ann McBurnie, William M. Vollmer, Suzanne Gillespie, Sean Sullivan, Todd A. Lee, Kevin B. Weiss, Robert L. Jensen, Robert Crapo, Paul Enright, David M. Mannino, John Cain, Rebecca Copeland, Dana Hazen, Jennifer Methvin, Vanessa Garcia Larsen

**Affiliations:** a National Heart and Lung Institute, Imperial College London, London, UK; b Royal Papworth Hospital NHS FT, Cambridge, UK; c Respiratory Services, Royal Commission Health Services Program, Jubail, Saudi Arabia; d Guttmacher Institute, New York, USA; e Ibn El Jazzar Faculty of Medicine of Sousse, University of Sousse, Sousse, Tunisia; f Department of Medical Sciences: Respiratory, Allergy and Sleep Research, Uppsala University, Uppsala, Sweden; g Department of Clinical Science, Faculty of Medicine, University of Bergen, Bergen, Norway; h Vadu Rural Health Program, KEM Hospital Research Centre, Pune, India; i Department of Medical Sciences, Clinical Physiology, Uppsala University, Uppsala, Sweden; j Dr. D. Y. Patil Medical College, Hospital and Research Centre, India; k Université de Sousse, Faculté de Médecine de Sousse, LR19ES09, Sousse 4000, Tunisia; l Faculty of Medicine, University of Iceland, Reykjavík, Iceland; m Department of Sleep, Landspitali - The National University Hospital of Iceland, Reykjavik, Iceland; n The Epidemiological Laboratory, Khartoum, Sudan; o Department of Community Health Sciences, Aga Khan University, Karachi, Pakistan; p Sher-i-Kashmir Institute of Medical Sciences, Srinagar, J&K, India; q Obafemi Awolowo University, Ile-Ife, Osun, Nigeria; r Department of Respiratory Medicine JSS Medical College, JSSAHER, India; s RCSI and UCD Malaysia Campus, Penang, Malaysia; t Escola Superior de Tecnologia da Saúde de Lisboa, Politécnico de Lisboa (Lisbon School of Health Technology, Polytechnic of Lisbon), Lisbon, Portugal; u Pulmonology Department, Santa Maria Local Health Unit, Lisbon, Portugal; v Institute of Environmental Health, Lisbon Medical School, Lisbon University, Lisbon, Portugal; w University of Kentucky, Lexington, KY, USA; x COPD Foundation, Miami, FL, USA; y Faculté de Médecine et de Pharmacie et de Médecine dentaire Fès, Morocco; z Directeur du Centre de Médecine Universitaire du Sommeil et Spécialiste Pneumologie CHU Hassan II Fès, Morocco; aa Epidemiology and Research in Health Sciences Laboratory, Faculty of Medicine, Pharmacy and Dentistry, Sidi Mohamed Ben Abdillah University, Morocco; ab Hassan Il University Hospital Centre of Fez, Morocco; ac Centre for Evidence Based Medicine, 2nd Department of Internal Medicine, Jagiellonian University Medical College, Kraków, Poland; ad Clinical Sciences, Liverpool School of Tropical Medicine, Pembroke Place, Liverpool, UK; ae Maastricht University Medical Centre, Maastricht, the Netherlands; af Ludwig Boltzmann Institute for Lung Health, Vienna, Austria; ag King Saud bin Abdulaziz University for Health Sciences, Riyadh, Saudi Arabia; ah King Abdullah International Medical Research Centre, Riyadh, Saudi Arabia; ai University of Cambridge, Cambridge, UK; aj Liverpool University Hospitals NHS Foundation Trust, Liverpool, UK; ak Department of Pulmonary Medicine, Paracelsus Medical University, Salzburg, Austria; al Pulmonology Department, Centro Hospitalar Universitário de Lisboa Central, Lisboa, Portugal; am NOVA Medical School, Nova University Lisbon, Lisboa, Portugal; an NIHR Imperial Biomedical Research Centre, London

**Keywords:** Spirometry, Asthma, Epidemiology, COPD, Bronchodilator

## Abstract

**Background:**

Bronchodilator responsiveness testing is mainly used for diagnosing asthma. We aimed to investigate whether it is associated with progression to chronic airflow obstruction over time.

**Methods:**

The multinational Burden of Obstructive Lung Disease cohort study surveyed adults, aged 40 years and above, at baseline and followed them up after a mean of 9.1 years. Recruitment took place between January 2, 2003 and December 26, 2016. Follow-up measurements were collected between January 29, 2019 and October 24, 2021. On both occasions, study participants provided information on respiratory symptoms, health status and several environmental and lifestyle exposures. They also underwent pre- and post-bronchodilator spirometry. We defined bronchodilator responsiveness at baseline using the American Thoracic Society and European Respiratory Society (ATS/ERS) 2022 definition, and the presence of chronic airflow obstruction at follow-up as a post-bronchodilator forced expiratory volume in 1 s to forced vital capacity ratio (FEV_1_/FVC) less than the lower limit of normal. We used multi-level regression models to estimate the association between baseline bronchodilator responsiveness and incident chronic airflow obstruction. We stratified analyses by gender and performed a sensitivity analysis in never smokers.

**Findings:**

We analysed data from 3701 adults with 56% being women. Compared to those without bronchodilator responsiveness at baseline, those with bronchodilator responsiveness had 36% increased risk of developing chronic airflow obstruction (RR: 1.36, 95%CI 1.04, 1.80). This effect was stronger in women (RR: 1.45, 95%CI 1.09, 1.91) than men (RR: 1.07, 95%CI 0.51, 2.24). Never smokers with bronchodilator responsiveness also were at greater risk of incident chronic airflow obstruction (RR: 1.48, 95%CI 1.01, 2.20).

**Interpretation:**

Bronchodilator responsiveness appears to be a risk factor for incident chronic airflow obstruction. It is important that future studies in other large population-based cohorts replicate these findings.

**Funding:**

10.13039/501100000850National Heart and Lung Institute, 10.13039/501100000265UK Medical Research Council, and 10.13039/100010269Wellcome Trust.

**Research in context**

**Evidence before this study**

We searched PubMed and Web of Science from database inception to September 24th, 2024. Search terms included: (“bronchodilator responsiveness” OR “bronchodilator reversibility” OR “BDR”) AND (“chronic airflow obstruction” OR “fixed airflow obstruction” OR “COPD” OR “FEV_1_/FVC”). Of the 237 studies returned by the search, most relevant was a recently published study that investigated whether bronchodilator responsiveness (significant improvement in lung function after inhalation of bronchodilator medication) was a risk factor for COPD in a cohort of tobacco smokers with a greater than or equal to 20 pack-year history. It found that bronchodilator responsiveness was associated with greater odds of incident COPD, however, given the population was at higher risk of chronic airflow obstruction due to smoking history, it remains unknown whether bronchodilator responsiveness has value as an indicator of future chronic airflow obstruction in general populations, where the prevalence of smoking is lower.

**Added value of this study**

To our knowledge, this is the first population-based cohort study to investigate the association between bronchodilator responsiveness and incident chronic airflow obstruction. The global scale of this study enabled comparison of this association across world regions, stratified by gender and smoking status.

**Implications of all the available evidence**

Our study has shown that bronchodilator responsiveness, even in the presence of lung function within normal limits, might be a risk factor for incident chronic airflow obstruction. Furthermore, we have cautiously highlighted potential gender differences in this association, with the impact appearing greater in women than men.

## Introduction

Bronchodilator responsiveness is defined by a significant improvement in lung function after inhalation of a bronchodilator delivered via metered dose inhaler (MDI) or nebuliser.^1^ Together with the presence of a characteristic pattern of respiratory symptoms, including wheezing, dyspnoea, chest tightness, or cough, and a variable expiratory flow limitation, bronchodilator responsiveness makes up an important component of an asthma diagnosis.^2^ However, it is not specific to asthma, and population-based studies have shown the prevalence of bronchodilator responsiveness to be similar among those with asthma and chronic obstructive pulmonary disease (COPD).^3^

It is known that some individuals with asthma will go on to develop irreversible lung damage, which is characterised by airway remodelling and a chronic “fixed” airflow obstruction, that is associated with accelerated lung function decline and significant morbidity.^4^ Chronic airflow obstruction is also a key component of a COPD diagnosis,^5^ with an estimated global prevalence of 11.2% in men and 8.6% in women.^6^ It is defined by the presence of an FEV_1_/FVC ratio less than the lower limit of normal (LLN), that persists after inhalation of a bronchodilator. Despite bronchodilator responsiveness being common in both asthma and COPD, the association between bronchodilator responsiveness and subsequent progression to chronic airflow obstruction has only been previously investigated in a cohort with a significant smoking pack-year history. Fortis and colleagues,^7^ found that bronchodilator responsiveness was associated with greater odds of incident COPD using data from the SPIROMICS cohort study. However, it is difficult to extrapolate their findings to the general population, where the prevalence and intensity of smoking are lower.

Chronic airflow obstruction is a significant public health concern. Strategies for the identification of those at risk are lacking, despite promising evidence that early airflow obstruction can be detected using spirometry, even in those who have never smoked.^8^, ^9^, ^10^ We used longitudinal data from the multinational Burden of Obstructive Lung Disease (BOLD) study, to investigate the association between bronchodilator responsiveness and incident chronic airflow obstruction, and the subsequent predictive ability of any relationship found.

## Methods

### Study design and participants

The protocols for both phases of data collection of BOLD have been published previously.^11^^,^^12^ Baseline data collection took place between January 2, 2003 and December 26, 2016. Non-institutionalised adults ≥40 years of age were recruited from 41 municipalities across 34 countries. Site specific sampling strategies were implemented to recruit representative samples of the populations studied. Between January 29, 2019 and October 24, 2021, participants from 18 sites were followed-up. For the present study, participants were included if they had completed the study core questionnaire, had acceptable pre- and post-bronchodilator spirometry, and no evidence of chronic airflow obstruction at baseline, and had acceptable post-bronchodilator spirometry at follow-up.

### Ethics

Ethical approval was obtained by each site from the local ethics committee, and informed consent was obtained from every participant. The follow-up study was also approved by Imperial College London Research Ethics Committee (ref. 17IC4272). All sites followed good clinical practice and local ethics regulations.

### Procedures

Demographic data and information on respiratory symptoms, health status, and exposures were collected by trained fieldworkers who administered standardised questionnaires translated into the local language. Fieldworkers measured standing height and weight and assessed lung function using spirometry. Lung function, including FEV_1_, FVC, and FEV_1_/FVC, was measured using the ndd EasyOne Spirometer (ndd Medizintechnik AG, Zurich, Switzerland), before and 15 min after inhalation of 200 mcg salbutamol via MDI through a spacer. Spirograms were centrally reviewed and assigned a quality score based on acceptability and reproducibility criteria.^13^ Data for gender were self-reported by study participants in the core questionnaire, with the options of male or female.

### Bronchodilator responsiveness

We used two different definitions of bronchodilator responsiveness. For the primary analysis we used the ATS/ERS 2022 definition,^1^ defined as change of >10% relative to the predicted value for FEV_1_ or FVC. We also performed a secondary analysis using the previous ATS/ERS 2005 definition to allow comparison,^14^ defined as change in FEV_1_ or FVC ≥12% and ≥200 mL of the initial value.

### Outcomes

The primary outcome was the association of baseline bronchodilator responsiveness with incident chronic airflow obstruction at follow-up. Chronic airflow obstruction was defined as a post-bronchodilator FEV_1_/FVC less than the lower limit of normal (LLN) according to reference equations for European Americans from the Third US National Health and Nutrition Examination Survey (NHANES).^15^ This approach to reference equations is in line with previous BOLD publications.^6^^,^^16^, ^17^, ^18^ Secondary outcomes included investigating whether associations were modified by gender, smoking status, or regional differences.

### Statistics

At baseline, we estimated the prevalence of bronchodilator responsiveness. We also estimated the prevalence of ever having had a self-reported doctor diagnosis of asthma. We evaluated the concordance between both bronchodilator responsiveness definitions using the Cohen’s κ coefficient. We calculated the incidence rate of chronic airflow obstruction per 1000 person-years for bronchodilator responsiveness and for those with a self-reported history of asthma and compared them to a reference population with no self-reported asthma, no evidence of bronchodilator responsiveness, and no chronic airflow obstruction at baseline. For incidence rates, we calculated Jackknife confidence intervals due to the use of inverse probability weights. We stratified these analyses by gender and World Health Organisation (WHO) region.

To estimate the association between having bronchodilator responsiveness at baseline and chronic airflow obstruction at follow-up, we performed multi-level (mixed effects) modified Poisson regression analyses with robust variance estimation, and reported the risk ratio (RR) with 95% confidence intervals.^19^ We fitted the models with a random intercept with study site as the effect term, to account for clustering by study site, and a random slope with bronchodilator responsiveness as the effect term, to average the association of bronchodilator responsiveness with chronic airflow obstruction across sites. The joint distribution of the random effects was assumed multivariate normal. We checked the linearity assumption by calculating the residuals for each level of the model and plotting them against quantitative predictors. We also used multi-level linear regression to estimate the association between bronchodilator responsiveness and post-bronchodilator FEV_1_/FVC ratio as a continuous measure. We used a directed acyclic graph (DAG) to decide on potential confounders a priori (efigure 1). They had to be risk factors for bronchodilator responsiveness or chronic airflow obstruction or both. We adjusted for gender (man/woman), age (years), BMI (kg/m^2^), smoking status (never/former/current), pack-years of smoking, pre-bronchodilator FEV_1_/FVC ratio, and follow-up time. Pack-years were calculated by number of cigarettes smoked per day divided by 20 and multiplied by years of smoking. In addition, we performed stratified analyses by gender to investigate possible effect modification, and a sensitivity analysis on never smokers. We repeated the above analyses to assess the association of having a self-reported doctor diagnosis of asthma at baseline and chronic airflow obstruction at follow-up. Self-reported asthma was defined as an affirmative answer to the question “has a doctor or other health care provider ever told you that you have asthma, asthmatic bronchitis or allergic bronchitis?” in the core questionnaire.

We constructed receiver operating characteristic (ROC) curves and calculated the area under the curve (AUC) for both bronchodilator responsiveness, and a self-reported doctor diagnosis of asthma, to determine their relative sensitivity and specificity in predicting chronic airflow obstruction. We compared the AUCs as previously described.^20^ All analyses were conducted using inverse probability weights (IPWs) to account for missing data at follow-up, generated as described in the cohort profile.^12^ All results were considered significant if the p-value was below 0.05. Analyses were performed using Stata 17 (Stata Corp., College Station, TX, USA).

### Role of the funding source

The funders of the study had no role in study design, data collection, data analysis, data interpretation, or writing of the report.

## Results

**Fig. 1:**
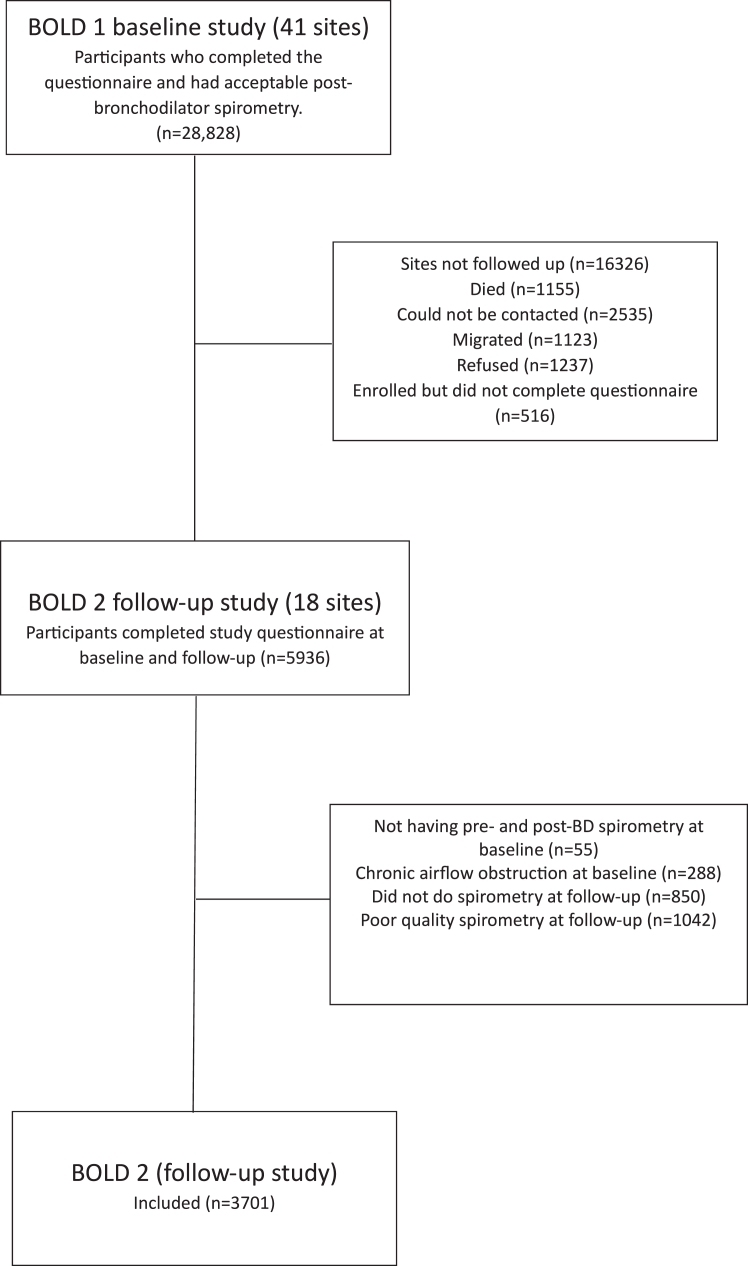
**Study flow diagram showing the inclusion and exclusion of participant data from Burden of Obstructive Lung Disease (BOLD) study**.

At baseline, 28,828 participants, across 41 sites, completed the core study questionnaire and had acceptable spirometry. Eighteen study sites took part in follow-up. Out of a possible 12,502 eligible participants, 1155 (9%) died, 2535 (20%) could not be contacted, 1123 (9%) migrated, 1237 (10%) refused to take part, and 516 (4%) enrolled but did not complete the core questionnaire. 5936 (48%) completed the core questionnaire. Of these, 3701 (62%) participants had acceptable pre- and post-bronchodilator spirometry and no evidence of chronic airflow obstruction at baseline, had acceptable post-bronchodilator spirometry at follow-up, and were included the present study (Fig. 1). Table 1 displays the baseline characteristics of the study population. There were more women than men (2078 of 3701, 56%). Mean (SD) age ranged from 45.8 years (6.4) in Mysore, India, to 58.2 years (10.2) in Tartu, Estonia. Mean (SD) BMI ranged from 21.8  kg m^−2^ (3.1) in Kashmir, India, to 30.8  kg m^−2^ (8.8) in Jamaica. Seventy-four percent (2725 of 3701) of the study population were never smokers.

**Table 1:** Baseline characteristics of study participants.

Total n = 3701	*n*	Women *n (%)*	Age, yrs Mean (SD)	BMI (kg·m^−2^) Mean (SD)	Never smoke *n (%)*	Bronchodilator responsiveness 2005 definition *n (%)*	Bronchodilator responsiveness 2022 definition *n (%)*	Asthma ever *n (%)*
Benin (Sémé-Kpodji)	131	63 (48%)	50.4 (7.8)	27.1 (5.6)	129 (98%)	6 (5%)	6 (5%)	1 (1%)
Estonia (Tartu)	176	89 (51%)	58.2 (10.2)	28.2 (4.8)	102 (58%)	9 (5%)	15 (9%)	10 (6%)
Iceland (Reykjavik)	253	123 (48%)	50.7 (7.9)	27.7 (4.6)	112 (44%)	10 (4%)	10 (4%)	41 (16%)
India (Kashmir)	43	17 (39%)	51.9 (10.0)	21.8 (3.1)	40 (93%)	0 (0%)	2 (5%)	0 (0%)
India (Mysore)	378	231 (61%)	45.8 (6.4)	24.8 (3.6)	355 (94%)	23 (6%)	15 (4%)	0 (0%)
India (Pune)	450	190 (42%)	50.2 (8.3)	22.5 (3.8)	410 (91%)	32 (7%)	30 (7%)	3 (1%)
Jamaica	22	9 (41%)	50.9 (6.4)	30.8 (8.8)	15 (58%)	0 (0%)	0 (0%)	2 (9%)
Kyrgyzstan (Chui)	308	223 (72%)	50.6 (7.7)	28.6 (5.5)	231 (75%)	23 (7%)	27 (8%)	8 (3%)
Kyrgyzstan (Naryn)	303	191 (63%)	49.6 (7.8)	26.9 (4.9)	242 (80%)	15 (5%)	20 (7%)	2 (1%)
Malawi (Chikwawa)	255	140 (55%)	52.9 (9.9)	22.0 (3.9)	188 (74%)	7 (3%)	11 (4%)	3 (1%)
Morocco (Fes)	16	5 (31%)	50.1 (5.2)	26.6 (5.1)	8 (50%)	0 (0%)	0 (0%)	0 (0%)
Nigeria (Ife)	363	258 (71%)	54.9 (11.3)	25.6 (5.4)	335 (92%)	34 (10%)	22 (6%)	0 (0%)
Norway (Bergen)	204	106 (52%)	53.9 (8.2)	26.1 (3.9)	72 (35%)	3 (1%)	8 (4%)	27 (13%)
Pakistan (Karachi)	183	110 (60%)	49.5 (8.3)	27.0 (5.5)	147 (80%)	12 (7%)	8 (4%)	3 (2%)
Philippines (Nampicuan-Talugtug)	248	135 (54%)	50.9 (8.3)	22.0 (4.4)	137 (55%)	15 (6%)	24 (10%)	12 (5%)
Sudan (Khartoum)	32	14 (44%)	50.8 (9.7)	27.9 (6.3)	20 (62%)	1 (3%)	1 (3%)	2 (6%)
Sweden (Uppsala)	185	89 (48%)	54.6 (8.0)	26.5 (3.7)	80 (43%)	7 (4%)	12 (7%)	25 (13%)
Tunisia (Sousse)	151	85 (56%)	51.8 (8.5)	30.2 (5.3)	102 (68%)	0 (0%)	5 (3%)	10 (7%)
Overall	3701	2078 (56%)	51.3 (9.0)	25.7 (5.2)	2725 (74%)	197 (5%)	216 (6%)	149 (4%)

Spirometry performed before and 15 min after inhalation of 200 mcg Salbutamol; responsiveness ATS/ERS 2005 definition: change in forced expiratory volume in 1 s (FEV_1_) or forced vital capacity (FVC) ≥12% and ≥200 mL of the initial value^14^; Reversibility ATS/ERS 2022 definition: change of >10% relative to the predicted value for FEV_1_ or FVC^1^; Asthma ever: If participants answered yes to “Has a doctor or other health care provider ever told you that you have asthma, asthmatic bronchitis or allergic bronchitis?” in the core questionnaire; Chronic airflow obstruction (CAO) defined if post-bronchodilator FEV_1_/FVC was less than the lower limit of normal (LLN) according to reference equations for European Americans in The Third National Health and Nutrition Survey (NHANES III).^15^

At baseline, 216 of 3701 (6%) had bronchodilator responsiveness according to the ATS/ERS 2022 definition. There were no cases of bronchodilator responsiveness in Jamaica and Fes (Morocco). Prevalence of bronchodilator responsiveness was highest in Nampicuan-Talugtug, Philippines (24 of 248, 10%). The prevalence of bronchodilator responsiveness for the ATS/ERS 2005 definition was 5% (197 of 3701), with a κ coefficient of 0.69 (95%CI 0.65, 0.73) indicating substantial agreement between the two definitions of bronchodilator responsiveness (Supplementary eTable S1, appendix). The prevalence of ever having had a doctor diagnosis of asthma was 4% (149 of 3701). There were no cases in Kashmir and Mysore (India), Fes (Morocco), and Ife (Nigeria). A doctor diagnosis of asthma was most prevalent in Reykjavik, Iceland (41 of 253, 16%). The κ coefficient for agreement between a doctor diagnosis of asthma and bronchodilator responsiveness was 0.02, indicating no agreement (Supplementary eTable S1, appendix).

**Fig. 2:**
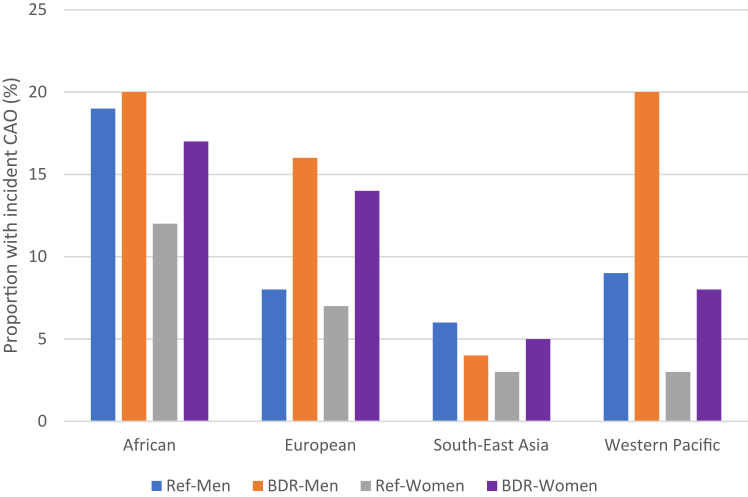
**Proportion progressing to chronic airflow obstruction by gender and WHO region.** Ref: Reference population with no self-reported asthma and no evidence of bronchodilator responsiveness and no chronic airflow obstruction (CAO) at baseline; BDR: responsiveness according to ATS/ERS 2022 definition-change of >10% relative to the predicted value for FEV_1_ or FVC. Chronic airflow obstruction defined if post-bronchodilator FEV_1_/FVC was less than the lower limit of normal (LLN) according to reference equations for European Americans in The Third National Health and Nutrition Survey (NHANES III).^15^ African region includes: Nigeria, Benin, Malawi. European region includes: Estonia, Iceland, Kyrgyzstan, Norway, and Sweden. South-East Asia includes: Indian sites. Western Pacific includes: Philippines.

The mean (SD) follow-up time was 9.1 years (3.3). Follow-up time was shortest in Karachi, Pakistan (4.4 years, 0.4) and longest in Bergen, Norway (15.1 years, 0.8). At follow-up, incident chronic airflow obstruction was 8% (297 of 3701). It was least common in Fes, Morocco (0%) and most common in Sémé-Kpodji, Benin (44 of 131, 34%) (Supplementary eTable S2, appendix). Of those with no bronchodilator responsiveness and no self-reported asthma as baseline, 253 of 3291 (8%) developed chronic airflow obstruction at follow-up. For those with bronchodilator responsiveness, 28 of 216 (13%) progressed to chronic airflow obstruction. Generally, participants with bronchodilator responsiveness who developed chronic airflow obstruction had fewer years of schooling, more years working a dusty job, and more self-reported dyspnoea and wheeze than those with bronchodilator responsiveness who did not develop chronic airflow obstruction (Supplementary eTable S3, appendix). Compared to those with no bronchodilator responsiveness and no self-reported asthma as baseline, proportionately more women than men with bronchodilator responsiveness progressed to chronic airflow obstruction overtime. This was seen across multiple world regions except for the European sites (Fig. 2). In participants with a self-reported doctor diagnosis of asthma, 14 of 149 (9%) progressed to chronic airflow obstruction.

**Fig. 3:**
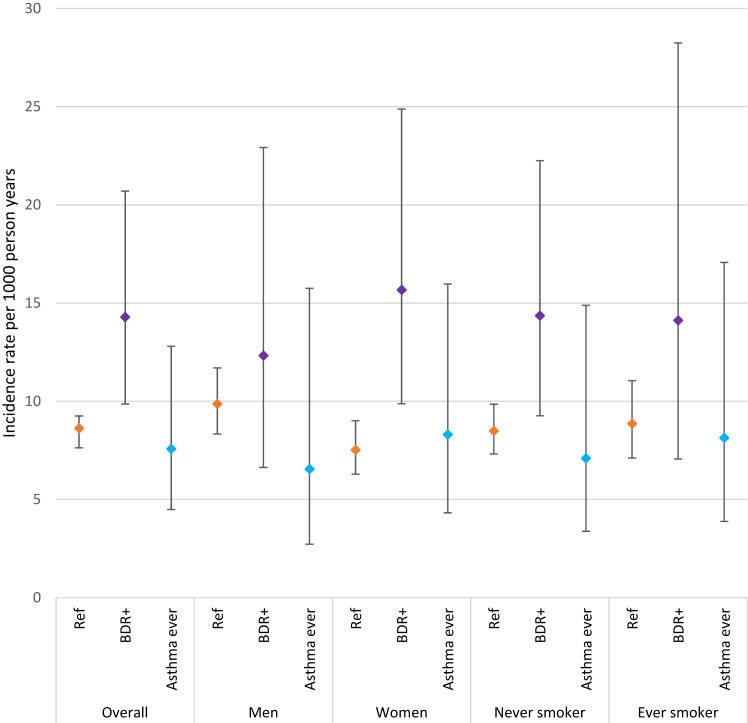
**Incidence rates of chronic airflow obstruction per 1000 person-years.** Error bars represent 95% confidence interval. Ref: Reference population with no self-reported asthma and no evidence of bronchodilator responsiveness and no chronic airflow obstruction at baseline; BDR+: Bronchodilator Responsiveness ATS/ERS 2022 definition: change of >10% relative to the predicted value for FEV_1_ or FVC^1^; Asthma ever: if participants answered yes to “Has a doctor or other health care provider ever told you that you have asthma, asthmatic bronchitis or allergic bronchitis?” in the core questionnaire Chronic airflow obstruction defined if post-bronchodilator FEV_1_/FVC was less than the lower limit of normal (LLN) according to reference equations for European Americans in The Third National Health and Nutrition Survey (NHANES III).^15^

The incidence rates of progression to chronic airflow obstruction per 1000 person-years are displayed in Fig. 3. Of those with no bronchodilator responsiveness and no self-reported asthma at baseline, the incidence rate of chronic airflow obstruction was 8.63 per 1000 person-years (95%CI, 7.63–9.25). The incidence rate of chronic airflow obstruction was higher for those with bronchodilator responsiveness at baseline (14.29 per 1000 person-years, 95%CI 9.86–20.70). For those with bronchodilator responsiveness, incidence of chronic airflow obstruction was higher among women compared to men and among never smokers compared to ever smokers (Fig. 1). Ever smoking was more common among men (693 of 1623, 43%) than women (283 of 2078, 13%). When stratifying by WHO region (Supplementary eFig. S2, appendix), incidence of chronic airflow obstruction was highest in the African region and lowest in South-East Asia. Except the African region, those with bronchodilator responsiveness at baseline generally had a higher incidence of chronic airflow obstruction than those in the reference population.

Compared to those with no bronchodilator responsiveness or a self-reported doctor diagnosis of asthma at baseline, those with bronchodilator responsiveness had a lower FEV_1_/FVC at follow-up (β: −1.76, 95%CI -2.74, −0.77) and greater risk of progressing to chronic airflow obstruction (RR: 1.36, 95%CI 1.04, 1.80). When stratifying by gender, women with bronchodilator responsiveness were more likely to progress to chronic airflow obstruction (RR: 1.45, 95%CI 1.09, 1.91) than men (RR: 1.07, 95%CI 0.51, 2.24). A similar association was seen in never smokers (RR: 1.48, 95%CI 1.01, 2.20) (Table 2). The results did not materially differ when using the ATS/ERS 2005 definition of bronchodilator responsiveness (Supplementary eTable S4). There was a suggestion of association between self-reported doctor diagnosis of asthma and progression to chronic airflow obstruction, particularly in women. This was also associated with having a lower FEV_1_/FVC at follow-up (Supplementary eTable S5). The AUC for the two models to discriminate incident chronic airflow obstruction were 0.76 (95%CI 0.73–0.79) for bronchodilator responsiveness and 0.73 (95%CI 0.70–0.76) for a self-reported doctor diagnosis of asthma (Supplementary eFig. S3, appendix).

**Table 2:** Association between bronchodilator responsiveness according to ATS/ERS 2022 criteria and incident chronic airflow obstruction.

	Total *n*	Bronchodilator Responsiveness ATS/ERS 2022 *n (%)*	CAO (follow-up) *n (%)*	RR (95%CI)	p-value	β coefficient (95%CI)^a^	p-value
Overall model	3634	216 (6%)	28 (13%)	1.36 (1.04, 1.80)	0.029	−1.76 (−2.74, −0.77)	<0.0001
Stratified by gender							
Men	1578	79 (5%)	10 (13%)	1.07 (0.51, 2.24)	0.85	−0.82 (−2.59, 0.95)	0.36
Women	2061	137 (7%)	18 (13%)	1.45 (1.09, 1.91)	0.010	−1.83 (−2.71, −0.95)	<0.0001
Never smoked	2688	163 (6%)	20 (12%)	1.48 (1.01, 2.20)	0.047	−1.67 (−3.47, 0.13)	0.069

Reference population are those without chronic airflow obstruction, bronchodilator responsiveness, or self-reported asthma at baseline. Linear associations between having bronchodilator responsiveness according to the ATS/ERS 2022 definition and follow-up post-bronchodilator FEV_1_/FVC ratio were estimated using mixed effects linear regression models.

a Negative regression coefficient indicates a reduction in FEV_1_/FVC ratio (i.e., worsened lung function). Associations between having bronchodilator responsiveness according to the ATS/ERS 2022 definition and progression to chronic airflow obstruction (CAO) were estimated using mixed effects Poisson regression models with robust variance estimation. Models were adjusted for gender, age, BMI, smoking status, smoking pack years, baseline FEV_1_/FVC, and follow-up time. As we expected associations to vary by study site, we fitted a random intercept to account for clustering by site and a random slope to average the associations across study sites. Model fitted with 16 clusters. Fes Morocco and Jamaica not included due to having an insufficient number of cases of bronchodilator responsiveness.

## Discussion

To the best of our knowledge, this is the first study to investigate the association between bronchodilator responsiveness and incident chronic airflow obstruction in the general population. We have shown that having bronchodilator responsiveness is associated with having lower lung function and increased risk of developing chronic airflow obstruction over time. This association was strongest in women and was also present in never smokers.

Overall, we found that having bronchodilator responsiveness at baseline was associated with 36% greater risk of progressing to chronic airflow obstruction over time, compared to not having bronchodilator responsiveness. Only one previous study has investigated this association in adults. Fortis and colleagues used data from nearly 1500 participants of the SPIROMICS study with normal baseline spirometry,^7^ and found that those with bronchodilator responsiveness had between 3 and 9.5 times greater odds of progressing to spirometrically defined COPD than those without bronchodilator responsiveness, depending on whether the bronchodilator responsiveness was inconsistent or consistent across repeated measurement visits. The effect seen in their study was greater than in ours. There are several possible reasons for this, first, the SPIROMICS cohort study is not representative of the general population, only recruiting participants with at least 20 pack year history of tobacco smoking. This means that participants were more likely to progress to COPD than in our study, where three-quarters of participants were never smokers.^6^ Secondly, the smoking effect was likely compounded by the older age of the cohort, with the average age in the SPIROMICS study being 63 years compared to 50 years in the present study.^21^ Finally, the definition of spirometrically defined COPD was different to our study, with Fortis and colleagues using the fixed cut-off of 0.7 for the FEV_1_/FVC. This approach has been shown to overestimate the prevalence of chronic airflow obstruction, especially in older age groups.^22^ Despite the differences in effect size, we provide further evidence from a general population sample that bronchodilator responsiveness is a risk factor for chronic airflow obstruction. Not all studies support this association. A study by Tantisira and colleagues found, in over 1000 asthmatic children, that grater bronchodilator responsiveness at baseline was associated with having a higher FEV_1_ percent predicted after 4 years of follow-up.^23^ The difference from our study is likely because of age, adults with asthma are more likely to develop fixed airflow obstruction due to the natural aging of the lung and increased likelihood of poor asthma management.^24^^,^^25^

We also found that women with bronchodilator responsiveness at baseline have lower lung function at follow-up and are 45% more likely to progress to chronic airflow obstruction over time, than women without bronchodilator responsiveness. We did not see the same association in men. There are no gender stratified studies to provide comparison, however, despite smoking being the strongest risk factor for chronic airflow obstruction,^6^ it is unlikely that it mediates this relationship, as the prevalence of smoking among women was only 13% compared to 43% of men. A more likely explanation is the role gender differences play in chronic respiratory disease. It is known that in adulthood, the prevalence and severity of asthma in women is greater than that of men,^26^ and similar is seen in COPD.^27^ In asthma, adult women have more rapid lung function decline and greater mortality than men.^28^ It is likely that some of this is explained by sex hormones. Takeda et al. showed, in mice following an ovalbumin challenge,^29^ that women had greater TH-2 inflammation and airway remodelling than men. Subsequent studies have shown that oestrogen signalling enhances TH-2 inflammation,^30^ while testosterone modulates it.^31^ Similar has also been seen in COPD, where animal studies have shown a relationship between oestrogen receptors and increased damage to the small airways after tobacco smoke exposure.^32^ It is also possible that socioeconomic status plays a role, as a previous analysis of data from the BOLD study has shown that use of respiratory medication and influenza vaccination is lower among sites from low-middle income countries.^33^ Furthermore, in the present study, the main difference between men and women was also seen in world regions with low-middle income sites. This supports previous research showing that women but not men from lower income backgrounds are at greater risk of asthma and asthmatic wheeze, although the exact causal mechanisms are not clear.^34^ Together with our results, this highlights the importance of greater awareness among doctors and healthcare professionals of the gender differences in chronic respiratory disease, especially for optimised disease management where bronchodilator responsiveness is present to prevent further lung function decline and the development of a chronic “fixed” airflow obstruction.^4^

We found that there was no agreement between a self-reported doctor diagnosis of asthma and either definition of bronchodilator responsiveness. This was not entirely unexpected, as previous population-based studies have shown that the prevalence of bronchodilator responsiveness in asthma and COPD is less than 20%.^3^ It is also likely that asthma is underdiagnosed in certain countries,^35^ for example, the BOLD site of Ife in Nigeria had the highest prevalence of bronchodilator responsiveness at 10%, but 0% reported a doctor diagnosis of asthma. This discordance highlights the importance of access to healthcare with appropriate diagnostic equipment, as the 10% with bronchodilator responsiveness would likely go undetected. While a self-reported doctor diagnosis of asthma was not significantly associated with incident chronic airflow obstruction in the analysis, there was a suggestion of an effect, which was supported by our finding that it was also associated with having a lower FEV_1_/FVC at follow-up.

When comparing the predictive abilities of the two models, bronchodilator responsiveness performed better than a doctor diagnosis of asthma in discriminating incident chronic airflow obstruction. This is in keeping with a previous study where we showed that spirometry was a better predictor of incident chronic airflow obstruction than self-reported respiratory symptoms, including respiratory wheeze.^10^ A study by Tan and colleagues in the Tasmanian Longitudinal Health Study found similar results,^8^ highlighting the limitations of self-reported metrics, where recall bias is common,^36^ over a tangible physiological measurement such as spirometry.

Our study has several strengths. First, its large sample size and population-based design make the results transferable to general populations. Spirometry was conducted by trained and certified technicians and lung function data were quality assured centrally. We also used both bronchodilator responsiveness definitions to enable direct comparison of their predictive ability. Our study also has limitations. The observational study design limits our ability to account for unmeasured confounding. The longitudinal component of this study was impacted by significant loss to follow-up caused by the COVID-19 pandemic. Although we attempted to account for this by using IPWs, it is possible that those present at follow-up are not entirely representative of the general population. We were also limited by sample size at site level, especially where prevalence of bronchodilator responsiveness at baseline and chronic airflow obstruction at follow-up was low, which restricted our ability to perform stratified analyses by world region. The smaller sample of men in some study sites may have also led to the lower precision of the risk estimate among this group. Finally, as follow-up periods varied considerably, it is possible that for some sites follow-up duration was insufficient for some with bronchodilator responsiveness to develop chronic airflow obstruction.

In conclusion, we have shown that bronchodilator responsiveness is a risk factor for chronic airflow obstruction, an association that appears stronger in women than men. It is important that future studies in other large population-based cohorts replicate these findings, and that work is done to elucidate the mechanisms behind this association.

## Contributors

BKB and AFSA conceived the study. BKB and FA performed data analysis and prepared the first drafts with input from AFSA. JPotts assisted with the preparation of the database. All authors contributed to further drafting, and read and approved the final version of the manuscript. BKB, FA, AFSA and JPotts had access to and verified the underlying data.

## Data sharing statement

De-identified participant data and questionnaires may be shared, after publication, on a collaborative basis upon reasonable request made to Dr Amaral (a.amaral@imperial.ac.uk). Requesting researchers will be required to submit an analysis plan.

## Declaration of interests

DM declares being a consultant to and receiving royalties from GlaxoSmithKline, AstraZeneca, and the COPD Foundation (royalty payments are up to date) and acting as an expert witness for Schlesinger Law Firm, outside of the submitted work. RN reports grants and personal fees from AstraZeneca and GlaxoSmithKline and grants from Boehringer Ingelheim and Novartis, outside of the submitted work. FR reports grants and personal fees from A. Menarini, Boehringer Ingelheim, Teva Pharma, Novartis, GlaxoSmithKline, AstraZeneca, VitalAire and Nippon Gases outside the submitted work. FF reports consulting fees from Sanofi and MSD, as well as personal and institutional fees from Sanofi, AstraZeneca, Chiesi, GSK and Pfizer, outside of the submitted work. AA reports research grants from the COLT foundation outside of the submitted work. All other authors declare no competing interests.

## Appendix ASupplementary data

Supplementary eFigs. S1–S3 and eTables S1–S5
