# Supplementary material for: Bronchodilator responsiveness and future chronic airflow obstruction: a multinational longitudinal study
Source: eClinicalMedicine. 2025 Feb 21;81:103123. doi: 10.1016/j.eclinm.2025.103123 (PMC11905823; doi:10.1016/j.eclinm.2025.103123)
Supplement: Supplementary eFigs. S1–S3 and eTables S1–S5 [file mmc1.docx]

**Supplemental appendix and BOLD group members**

**Contents**

[**eTable 1. Level of agreement between ATS/ERS 2005 and 2022 bronchodilator responsiveness definitions and self-reported doctor diagnosis of asthma. 2**](#_Toc185447229)

[**eTable 2. Follow-up time and incident chronic airflow obstruction 3**](#_Toc185447230)

[**eFigure 1. Causal diagram for the association between bronchodilator responsiveness and chronic airflow obstruction. 4**](#_Toc185447231)

[**eFigure 2. Incidence rates of chronic airflow obstruction per 1000 person-years by WHO region. 5**](#_Toc185447232)

[**eTable 3. Baseline characteristics of those with bronchodilator responsiveness who developed chronic airflow obstruction vs those who did not 6**](#_Toc185447233)

[**eTable 4. Association between bronchodilator responsiveness according to ATS/ERS 2005 criteria and incident chronic airflow obstruction. 7**](#_Toc185447234)

[**eTable 5. Association between ever having had a self-reported doctor diagnosis of asthma and incident chronic airflow obstruction. 8**](#_Toc185447235)

[**eFigure 3. Receiver operating characteristic curve (ROC) and area under the curve (AUC) for the ability of bronchodilator (BDR) responsiveness and self-reported asthma to discriminate incident chronic airflow obstruction. 9**](#_Toc185447236)

[**BOLD (Burden of Obstructive Lung Disease) Collaborative Research Group members 10**](#_Toc185447237)

[**References 12**](#_Toc185447238)

# **eTable 1. Level of agreement between ATS/ERS 2005 and 2022 bronchodilator responsiveness definitions and self-reported doctor diagnosis of asthma.**

|  | **Agreement (%)** | **Expected agreement (%)** | **Kappa (95%CI)** |
| --- | --- | --- | --- |
| ATS/ERS 2005 vs ATS/ERS 2022 | 96·73 | 89·46 | 0·69 (0·65, 0·73) |
| ATS/ERS 2005 vs Asthma ever | 91·17 | 91·06 | 0·01 (-0·03, 0·05) |
| ATS/ERS 2022 vs Asthma ever | 90·82 | 90·59 | 0·02 (-0·02, 0·06) |

Level of agreement classified according to Landis and Koch (3), below 0·0= poor, 0·00-0·2=slight, 0·21-0·40= fair, 0·41-0·60= moderate, 0·61-0·80= substantial, >0·81= almost perfect. Responsiveness ATS/ERS 2005 definition: change in forced expiratory volume in 1 second (FEV_1_) or forced vital capacity (FVC) ≥12% and ≥200mL of the initial value (4); Responsiveness ATS/ERS 2022 definition: change of >10% relative to the predicted value for FEV_1_ or FVC (1); Asthma ever: Answered yes to “has a doctor or healthcare professional ever diagnosed you with asthma”;

| **Total n = 3701** | ***n*** | **Follow-up time, yrs,**  ***mean (SD)*** | **CAO at follow-up, *n (%)*** |
| --- | --- | --- | --- |
| Benin (Sémé-Kpodji) | 131 | 7·0 (0·2) | 44 (34%) |
| Estonia (Tartu) | 176 | 10·8 (0·7) | 13 (7%) |
| Iceland (Reykjavik) | 253 | 14·7 (0·5) | 14 (5%) |
| India (Kashmir) | 43 | 8·4 (0·1) | 10 (23%) |
| India (Mysore) | 378 | 7·2 (1·0) | 19 (5%) |
| India (Pune) | 450 | 10·9 (0·3) | 11 (2%) |
| Jamaica | 22 | 5·4 (0·2) | 1 (5%) |
| Kyrgyzstan (Chui) | 308 | 6·1 (0·1) | 48 (16%) |
| Kyrgyzstan (Naryn) | 303 | 6·1 (0·1) | 9 (3%) |
| Malawi (Chikwawa) | 255 | 4·8 (0·4) | 22 (9%) |
| Morocco (Fes) | 16 | 10·5 (0·3) | 0 (0%) |
| Nigeria (Ife) | 363 | 8·3 (0·6) | 45 (12%) |
| Norway (Bergen) | 204 | 15·1 (0·8) | 12 (6%) |
| Pakistan (Karachi) | 183 | 4·4 (0·4) | 3 (2%) |
| Philippines (Nampicuan-Talugtug) | 248 | 10·8 (0·3) | 18 (7%) |
| Sudan (Khartoum) | 32 | 7·8 (0·4) | 2 (6%) |
| Sweden (Uppsala) | 185 | 13·4 (0·5) | 12 (7%) |
| Tunisia (Sousse) | 151 | 10·4 (0·5) | 14 (9%) |
| Overall | 3701 | 9·1 (3·3) | 297 (8%) |

# **eTable 2. Follow-up time and incident chronic airflow obstruction**

Chronic airflow obstruction (CAO) defined if post-bronchodilator FEV_1_/FVC was less than the lower limit of normal (LLN) according to reference equations for European Americans in The Third National Health and Nutrition Survey (NHANES III) (2)

*
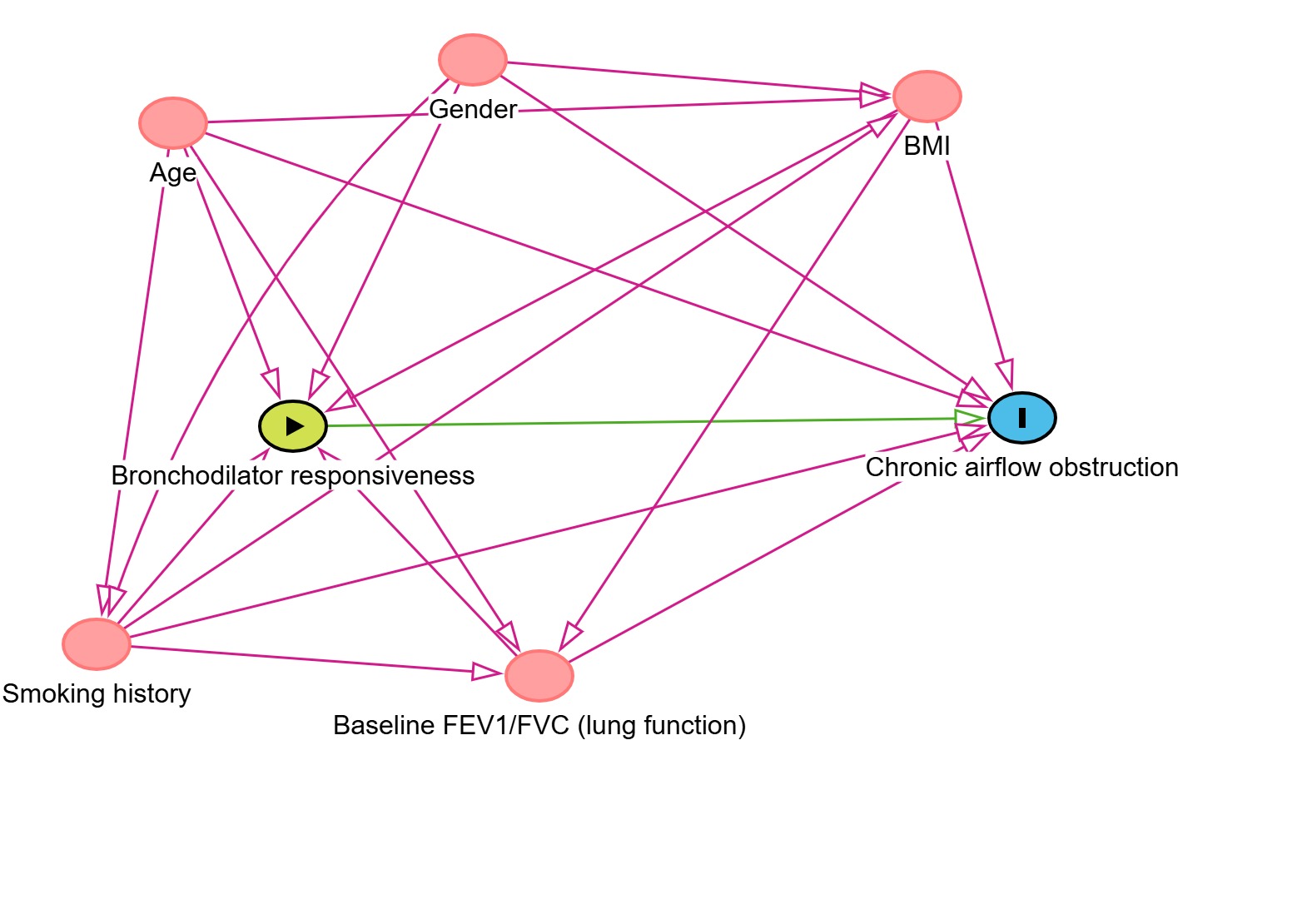
*

# **eFigure 1. Causal diagram for the association between bronchodilator responsiveness and chronic airflow obstruction.**

**Rationale:** Age is a known risk factor for both asthma and COPD and is therefore associated with bronchodilator responsiveness and chronic airflow obstruction (5, 6); High BMI is a risk factor for asthma and low BMI is a risk factor for COPD (7, 8); Gender differences are seen in the prevalence and severity of asthma and COPD (5), with women more likely to have asthma, and to have more severe asthma, predisposing to greater airflow obstruction (9); Smoking is associated with both asthma and COPD, and is the primary risk factor for chronic airflow obstruction (5, 7); Lower baseline lung function increases the likelihood of bronchodilator responsiveness (10), lower lung function is also associated with progression to more established airflow obstruction over time (11, 12).

# **eFigure 2. Incidence rates of chronic airflow obstruction per 1000 person-years by WHO region.**

Error bars represent 95% confidence interval. WHO: world health organisation; Ref: Reference population with no self-reported asthma and no evidence of bronchodilator responsiveness; BDR+: Bronchodilator responsiveness ATS/ERS 2022 definition: change of >10% relative to the predicted value for FEV_1_ or FVC (1); Asthma ever: Answered yes to “has a doctor or healthcare professional ever diagnosed you with asthma”; Chronic airflow obstruction (CAO) defined if post-bronchodilator FEV_1_/FVC was less than the lower limit of normal (LLN) according to reference equations for European Americans in The Third National Health and Nutrition Survey (NHANES III) (2). American and Eastern Mediterranean regions excluded due to having insufficient cases of bronchodilator Responsiveness for either definition. African region includes: Nigeria, Benin, Malawi. European region includes: Estonia, Iceland, Kyrgyzstan, Norway, and Sweden. South-East Asia includes: Indian sites. Western Pacific includes: Philippines.

# **eTable 3. Baseline characteristics of those with bronchodilator responsiveness who developed chronic airflow obstruction vs those who did not**

|  | **Bronchodilator responsiveness with no incident CAO**  **(n=188)** | **Bronchodilator responsiveness with incident CAO**  **(n=28)** |
| --- | --- | --- |
| **Age, mean (SD)** | 55·1 (10·7) | 58·0 (9·4) |
| **Women, n (%)** | 119 (63%) | 18 (64%) |
| **Follow-up time, years, mean (SD)** | 9·1 (3·1) | 8·9 (2·9) |
| **BMI kg·m^−2^, mean (SD)** | 25·6 (5·1) | 26·2 (5·9) |
| **Smoking status** |  |  |
| Never, n (%) | 141 (75%) | 20 (71%) |
| Former, n (%) | 23 (12%) | 4 (14%) |
| Current, n (%) | 24 (13%) | 4 (14%) |
| Pack years, mean (SD) | 3·7 (9·4) | 3·5 (9·3) |
| **Exposures** |  |  |
| Family history of COPD, n (%) | 14 (8%) | 0 (0%) |
| Passive smoke exposure, n (%) | 24 (13%) | 1 (4%) |
| Childhood hospitalisation for breathing, n (%) | 6 (3%) | 1 (4%) |
| Previous tuberculosis, n (%) | 6 (3%) | 0 (0%) |
| Years of schooling, mean (SD) | 8·9 (5·7) | 7·0 (5·3) |
| Years working in a dusty job, mean (SD) | 3·8 (9·9) | 5·2 (10·3) |
| **Symptoms** |  |  |
| Dyspnoea, n (%) | 31 (17%) | 9 (32%) |
| Chronic cough, n (%) | 17 (9%) | 3 (11%) |
| Chronic phlegm, n (%) | 9 (5%) | 2 (7%) |
| Wheeze, n (%) | 24 (13%) | 5 (18%) |
| **Spirometry** |  |  |
| Post-BD FEV_1_/FVC %, mean (SD) | 78·5 (5·9) | 73·7 (6·0) |
| Post-BD FEV_1_ Litres, mean (SD) | 2·5 (0·7) | 2·4 (0·8) |
| Post-BD FEF_25-75_ L/min, mean (SD) | 2·4 (1·0) | 1·8 (0·6) |
| Post-BD FVC Litres, mean (SD) | 3·2 (1·0) | 3·3 (1·3) |

Bronchodilator responsiveness ATS/ERS 2022 definition: change of >10% relative to the predicted value for FEV_1_ or FVC (1); Dyspnoea measured according to mMRC Dyspnoea scale: 0-1= minimal/no breathlessness, ≥2= significant breathlessness. Chronic cough: cough on most days for 3 months each year. Chronic Phlegm: Phlegm on most days three months each year. Wheeze: Wheezing or whistling in the chest at any time in the last 12 months; passive smoking: whether somebody else smoked in the participant's home in the past 2 weeks; Family history of COPD: mother, father, sister, or brother ever diagnosed with emphysema, chronic bronchitis, or chronic obstructive pulmonary disease by a doctor; Previous Tuberculosis: Previous Tuberculosis diagnosed by a doctor FEV_1_/FVC: Forced expiratory volume in one second as a ratio of the forced vital capacity; FEF_25-75_: Mean forced expiratory flow rate between 25% and 75% of the forced vital capacity; Family history of COPD: BD: Bronchodilator – 200mcg Salbutamol.

# **eTable 4. Association between bronchodilator responsiveness according to ATS/ERS 2005 criteria and incident chronic airflow obstruction.**

|  | **Total**  ***n*** | **Responsiveness**  **ATS/ERS 2005**  ***n (%)*** | **CAO**  **(follow-up)**  ***n (%)*** | **RR** **(95%CI)** | **p-value** | **β coefficient**  **(95%CI)^*^** | **p-value** |
| --- | --- | --- | --- | --- | --- | --- | --- |
| **Overall model** | 3449 | 197 (6%) | 24 (12%) | 1·21 (0·83, 1·77) | 0·31 | -1·60 (-2·91, -0·29) | 0·017 |
| **Stratified by gender** |  |  |  |  |  |  |  |
| Men | 1486 | 72 (5%) | 8 (11%) | 1·03 (0·53, 1·98) | 0·94 | -0·86 (-2·22, 0·48) | 0·21 |
| Women | 1954 | 125 (6%) | 16 (13%) | 1·32 (0·99, 1·76) | 0·063 | -2·28 (-4·25, -0·31) | 0·023 |
| **Never smoked** | 2546 | 162 (6%) | 20 (12%) | 1·54 (0·90, 2·64) | 0·12 | -1·62 (-3·26, 0·01) | 0·052 |

Reference population are those without chronic airflow obstruction, bronchodilator responsiveness, or self-reported asthma at baseline. Linear associations between having bronchodilator responsiveness according to the ATS/ERS 2005 definition and follow-up post-bronchodilator FEV_1_/FVC ratio were estimated using mixed effects linear regression models. *Negative regression coefficient indicates a reduction in FEV_1_/FVC ratio (i.e., worsened lung function). Associations between having bronchodilator responsiveness according to the ATS/ERS 2005 definition and progression to chronic airflow obstruction (CAO) were estimated using mixed effects Poisson regression models with robust variance estimation. Models were adjusted for gende**r**, age, BMI, smoking status, smoking pack years, baseline FEV_1_/FVC, and follow-up time. As we expected associations to vary by study site, we fitted a random intercept to account for clustering by site and a random slope to average the associations across study sites. Model fitted with 14 clusters. Sousse Tunisia, Kashmir India, Fes Morocco, and Jamaica not included due to having an insufficient number of cases of bronchodilator responsiveness.

# **eTable 5. Association between ever having had a self-reported doctor diagnosis of asthma and incident chronic airflow obstruction.**

|  | **Total**  ***n*** | **Asthma ever**  ***n (%)*** | **CAO**  **(follow-up)**  ***n (%)*** | **RR** **(95%CI)** | **p-value** | **β coefficient**  **(95%CI)^*^** | **p-value** |
| --- | --- | --- | --- | --- | --- | --- | --- |
| **Overall model** | 2874 | 149 (5%) | 14 (9%) | 1·15 (0·68, 1·95) | 0·59 | -2·38 (-3·38, -1·39) | <0·0001 |
| **Stratified by gender** |  |  |  |  |  |  |  |
| Men | 1314 | 58 (4%) | 5 (9%) | 0·85 (0·40, 1·82) | 0·67 | -3·46 (-4·65, -2·27) | <0·0001 |
| Women | 1560 | 91 (6%) | 9 (10%) | 1·43 (0·68, 3·02) | 0·35 | -1·79 (-3·02, -0·56) | 0·004 |
| **Never smoked** | 1974 | 86 (4%) | 7 (8%) | 1·17 (0·29, 4·72) | 0·82 | -1·77 (-3·77, 0·22) | 0·082 |

Reference population are those without chronic airflow obstruction, bronchodilator responsiveness, or self-reported asthma at baseline. Linear associations between having ever had a self-reported doctor diagnosis of asthma and follow-up post-bronchodilator FEV_1_/FVC ratio were estimated using mixed effects linear regression models. *Negative regression coefficient indicates a reduction in FEV_1_/FVC ratio (i.e., worsened lung function). Associations between having ever had a self-reported doctor diagnosis of asthma and progression to chronic airflow obstruction (CAO) were estimated using mixed effects Poisson regression models with robust variance estimation. Models were adjusted for gender, age, BMI, smoking status, smoking pack years, baseline FEV_1_/FVC and follow-up time. As we expected associations to vary by study site, we fitted a random intercept to account for clustering by site and a random slope to average the associations across study sites. Model fitted with 14 clusters. Mysore India, Kashmir India, Fes Morocco, and Ife Nigeria not included due to having an insufficient number of cases of cases of self-reported doctor diagnosis of asthma.

# **eFigure 3. Receiver operating characteristic curve (ROC) and area under the curve (AUC) for the ability of bronchodilator (BDR) responsiveness and self-reported asthma to discriminate incident chronic airflow obstruction.**

# **BOLD (Burden of Obstructive Lung Disease) Collaborative Research Group members**

**Albania**: Hasan Hafizi (principal investigator [PI]), Anila Aliko, Donika Bardhi, Holta Tafa, Natasha Thanasi, Arian Mezini, Alma Teferici, Dafina Todri, Jolanda Nikolla, and Rezarta Kazasi (Tirana University Hospital Shefqet Ndroqi, Albania); **Algeria**: Hamid Hacene Cherkaski (PI), Amira Bengrait, Tabarek Haddad, Ibtissem Zgaoula, Maamar Ghit, Abdelhamid Roubhia, Soumaya Boudra, Feryal Atoui, Randa Yakoubi, Rachid Benali (Department of Pneumology, Faculty of Medicine, Annaba, Algeria), Abdelghani Bencheikh and Nadia Ait-Khaled (Department of Epidemiology and Prevention, EPHS ElHadjar, Algeria); **Australia**: Christine Jenkins (PI), Guy Marks (PI), Tessa Bird, Paola Espinel, Kate Hardaker, Brett Toelle (Woolcock Institute of Medical Research, Sidney, Australia); **Austria**: Michael Studnicka (PI), Torkil Dawes, Bernd Lamprecht, and Lea Schirhofer (Department of Pulmonary Medicine, Paracelsus Medical University, Salzburg, Austria); **Benin**: Herve Lawin (PI), Arsene Kpangon, Karl Kpossou, Gildas Agodokpessi, Paul Ayelo, Benjamin Fayomi, Rolus Atrokpo, Gaston Hounton, Dieudonnè Yadjodo (Unit of Teaching and Research in Occupational and Environmental Health, University of Abomey Calavi, Cotonou, Benin); **Cameroon**: Bertrand Mbatchou (PI), Atongno Humphrey Ashu (Douala General Hospital, Douala, Cameroon); **Canada**: Wan C Tan (PI) and Wen Wang (iCapture Center for Cardiovascular and Pulmonary Research, University of British Columbia, Vancouver, BC, Canada); **China**: NanShan Zhong (Principal Investigator [PI]), Shengming Liu, Jiachun Lu, Pixin Ran, Dali Wang, Jin-ping Zheng, and Yumin Zhou (Guangzhou Institute of Respiratory Health, First Affiliated Hospital of Guangzhou Medical College, Guangzhou, China); **Estonia**: Rain Jõgi (PI), Hendrik Laja, Katrin Ulst, Vappu Zobel, Toomas-Julius Lill, Katrin Kiili, and Ira Laanelepp (Lung Clinic, Tartu University Hospital, Tartu, Estonia); **Germany**: Tobias Welte (PI), Isabelle Bodemann, Henning Geldmacher, and Alexandra Schweda-Linow (Dept of Pneumology, Hannover Medical School and German Center of Lung Research, Hannover, Germany); **Iceland**: Thorarinn Gislason (PI), Bryndis Benedikdtsdottir, Kristin Jörundsdottir, Lovisa Gudmundsdottir, Sigrun Gudmundsdottir, Gunnar Gudmundsson, Elin Helga Thorarinsdottir, and Hjördis Sigrun Pálsdottir (Department of Allergy, Respiratory Medicine, and Sleep, Landspitali University Hospital, Reykjavik, Iceland); **India**: Mahesh Padukudru Anand (PI) (JSS Medical College, JSSAHER, Mysuru, India); Parvaiz A Koul (PI), Sajjad Malik, Nissar A Hakim, and Umar Hafiz Khan (Sher-i-Kashmir Institute of Medical Sciences, Srinagar, J&K, India); Rohini Chowgule (PI), Vasant Shetye, Jonelle Raphael, Rosel Almeda, Mahesh Tawde, Rafiq Tadvi, Sunil Katkar, Milind Kadam, Rupesh Dhanawade, and Umesh Ghurup (Indian Institute of Environmental Medicine, Mumbai, India); Sanjay Juvekar (PI), Siddhi Hirve, Somnath Sambhudas, Bharat Chaidhary, Meera Tambe, Savita Pingale, Arati Umap, Archana Umap, Nitin Shelar, Sampada Devchakke, Sharda Chaudhary, Suvarna Bondre, Savita Walke, Ashleshsa Gawhane, Anil Sapkal, Rupali Argade, Vijay Gaikwad, Dhiraj Agrawal, Babu Pawar, Shalan Mhetre, Namdev Kale, and Shirish Kathale (Vadu Rural Health Program, Pune, India); Sundeep Salvi (PI), Bill Brashier, Jyoti Londhe, and Sapna Madas (Chest Research Foundation, Pune, India); **Jamaica**: Althea Aquart-Stewart (PI), Akosua Francia Aikman (University of the West Indies, Kingston, Jamaica); **Kyrgyzstan**: Talant M Sooronbaev (PI), Bermet M Estebesova, Meerim Akmatalieva, Saadat Usenbaeva, Jypara Kydyrova, Eliza Bostonova, Ulan Sheraliev, Nuridin Marajapov, Nurgul Toktogulova, Berik Emilov, Toktogul Azilova, Gulnara Beishekeeva, Nasyikat Dononbaeva, and AijamalTabyshova (Pulmunology and Allergology Department, National Centre of Cardiology and Internal Medicine, Bishkek, Kyrgyzstan); **Malawi**: Kevin Mortimer (Baseline PI), Wezzie Nyapigoti, Ernest Mwangoka, Mayamiko Kambwili, Martha Chipeta, Gloria Banda, Suzgo Mkandawire, Justice Banda, Graham Devereux (Follow-up PI), Jamie Rylance, Martin Njoroge, Catherine Chirwa, Chifundo Mhango, Edgar Ngwira, Faith Zumazuma, Frank Jonas, and Patrick Mjojo (the Malawi Liverpool Wellcome Trust, Blantyre, Malawi); **Malaysia**: Li-Cher Loh (PI), Abdul Rashid, and Siti Sholehah (Royal College of Surgeons in Ireland and University College Dublin Malaysia Campus (RUMC)); **Morocco**: Mohamed C Benjelloun (Baseline PI), Chakib Nejjari, Mohamed Elbiaze, Karima El Rhazi (Follow-up PI), Manelle Rjimati, Btissame ElHarche, Reda Benjelloun, and Yassin Chefchaou (Laboratoire d’épidémiologie, Recherche Clinique et Santé Communautaire, Fès, Morroco); **The Netherlands**: E F M Wouters and G J Wesseling (Maastricht University Medical Center, Maastricht, the Netherlands); **Nigeria**: Daniel Obaseki (PI), Gregory Erhabor, Olayemi Awopeju, and Olufemi Adewole (Obafemi Awolowo University, Ile-Ife, Nigeria); **Norway**: Amund Gulsvik (Baseline PI), Tina Endresen, Lene Svendsen (Department of Thoracic Medicine, Institute of Medicine, University of Bergen, Bergen, Norway), and Rune Nielsen (Follow-up PI), Marit Aardal, Hildegunn B Fleten, Gerd Eli Dale, Eli Nordeide, Malin P Grøttveit, Åsa Skjelde, Ane Aamli Gagnat, Anders Ørskov Rotevatn, Marta Erdal (Department of Clinical Science, University of Bergen, Bergen, Norway); **Pakistan**: Asaad A Nafees (PI), Muhammad Irfan, Hasan Nawaz Tahir, Muhammad Noman, Roman Ul Haq (Aga Khan Univeristy, Karachi, Pakistan); **Philippines**: Luisito F Idolor (Baseline PI), Teresita S de Guia, Norberto A Francisco, Camilo C Roa, Fernando G Ayuyao, Cecil Z Tady, Daniel T Tan, Sylvia Banal-Yang, Vincent M Balanag, Jr, Maria Teresita N Reyes, Renato B Dantes, and Stefanni Nonna M Paraguas (Follow-up PI) (Lung Centre of the Philippines and Philippine Heart Centre, Philippine General Hospital, Nampicuan and Talugtug, the Philippines); Renato B Dantes (Baseline PI), Lourdes Amarillo, Lakan U Berratio, Lenora C Fernandez, Norberto A Francisco, Gerard S Garcia, Teresita S de Guia, Luisito F Idolor, Sullian S Naval, Thessa Reyes, Camilo C Roa, Jr, Ma Flordeliza Sanchez, and Leander P Simpao (Philippine College of Chest Physicians, Manila, the Philippines); **Poland**: Ewa Nizankowska-Mogilnicka (PI), Jakub Frey, Rafal Harat, Filip Mejza, Pawel Nastalek, Andrzej Pajak, Wojciech Skucha, Andrzej Szczeklik, and Magda Twardowska, (Division of Pulmonary Diseases, Department of Medicine, Jagiellonian University School of Medicine, Krakow, Poland); **Portugal**: Cristina Bárbara (PI), Fátima Rodrigues, Hermínia Dias, João Cardoso, João Almeida, Maria João Matos, Paula Simão, Moutinho Santos, and Reis Ferreira (the Portuguese Society of Pneumology, Lisbon, Portugal); **Saudi Arabia**: M Al Ghobain (PI), H Alorainy (PI), E El-Hamad, M Al Hajjaj, A Hashi, R Dela, R Fanuncio, E Doloriel, I Marciano, and L Safia (Saudi Thoracic Society, Riyadh, Saudi Arabia); **South Africa**: Eric Bateman (Baseline PI), Anamika Jithoo (Baseline PI), Desiree Adams, Edward Barnes, Jasper Freeman, Anton Hayes, Sipho Hlengwa, Christine Johannisen, Mariana Koopman, Innocentia Louw, Ina Ludick, Alta Olckers, Johanna Ryck, Janita Storbeck, and Richard van Zyl-Smit (Follow-up PI) (University of Cape Town Lung Institute, Cape Town, South Africa); **Sri Lanka**: Kirthi Gunasekera (PI), Rajitha Wickremasinghe (Medical Research Institute, Central Chest Clinic, Colombo, Sri Lanka); **Sudan**: Asma Elsony (Baseline PI), Hana A Elsadig, Nada Bakery Osman, Bandar Salah Noory, Monjda Awad Mohamed, Hasab Alrasoul Akasha Ahmed Osman, Namarig Moham ed Elhassan, Abdel Mu‘is El Zain, Marwa Mohamed Mohamaden, Suhaiba Khalifa, Mahmoud Elhadi, Mohand Hassan, Dalia Abdelmonam, Rana Ahmed (Follow-up PI), Rashid Osman, Hind Eltigani, Najlaa Mohamed Abass, Ahmed Beriar Ahmed, Sahar AlaElddin (Epidemiological Laboratory, Khartoum, Sudan); **Sweden**: Christer Janson (PI), Inga Sif Olafsdottir, Katarina Nisser, Ulrike Spetz-Nyström, Gunilla Hägg, Gun-Marie Lund, Andrei Malinovschi, Eva Wallberg, Birgitta Appelfeldt, and Mona Andrén (Department of Medical Sciences: Respiratory Medicine and Allergology, Uppsala University, Uppsala, Sweden); **Trinidad and Tobago**: Terence Seemungal (PI), Fallon Lutchmansingh, Liane Conyette (University of the West Indies, St Augustine, Trinidad and Tobago); **Tunisia**: Imed Harrabi (Baseline PI), Myriam Denguezli (Follow-up PI), Zouhair Tabka (deceased), Hager Daldoul, Zaki Boukheroufa, Firas Chouikha, Wahbi Belhaj Khalifa, Safa Hsan, Nadia Lakhdar, and Mounir Landolsi (University Hospital Farhat Hached, Faculté de Médecine, Sousse, Tunisia); **Turkey**: Ali Kocabaş (PI), Attila Hancioglu, Ismail Hanta, Sedat Kuleci, Ahmet Sinan Turkyilmaz, Sema Umut, and Turgay Unalan (Department of Chest Diseases, Cukurova University School of Medicine, Adana, Turkey); **UK**: Peter G J Burney (Baseline and Follow-up PI), Anamika Jithoo, Louisa Gnatiuc, Hadia Azar, Jaymini Patel, Caron Amor, James Potts, Michael Tumilty, Fiona McLean, Risha Dudhaiya, Andre F S Amaral (Project lead), Octavia Mulhern, Emmanouil Bagkeris, Jasleen Gegic, Paul Cullinan, Cosetta Minelli (National Heart and Lung Institute, Imperial College London, London, UK); **USA**: A Sonia Buist (Baseline PI) (Oregon Health & Science University, Portland, OR), Mary Ann McBurnie, William M Vollmer, Suzanne Gillespie (Kaiser Permanente Center for Health Research, Portland, OR); Sean Sullivan (University of Washington, Seattle, WA); Todd A Lee, Kevin B Weiss, (Northwestern University, Chicago, IL); Robert L Jensen, Robert Crapo (Latter Day Saints Hospital, Salt Lake City, Utah); Paul Enright (University of Arizona, Tucson, AZ); David M Mannino (PI), John Cain, Rebecca Copeland, Dana Hazen, and Jennifer Methvin, (University of Kentucky, Lexington, KY); Vanessa Garcia Larsen (John Hopkins Bloomberg School of Public Health, Baltimore, MD).

# **References**

1. Stanojevic S, Kaminsky DA, Miller MR, Thompson B, Aliverti A, Barjaktarevic I, et al. ERS/ATS technical standard on interpretive strategies for routine lung function tests. Eur Respir J. 2022;60(1).

2. Hankinson JL, Odencrantz JR, Fedan KB. Spirometric reference values from a sample of the general U.S. population. Am J Respir Crit Care Med. 1999;159(1):179-87.

3. Landis JR, Koch GG. The measurement of observer agreement for categorical data. Biometrics. 1977;33(1):159-74.

4. Miller MR, Hankinson J, Brusasco V, Burgos F, Casaburi R, Coates A, et al. Standardisation of spirometry. Eur Respir J. 2005;26(2):319-38.

5. Burney P, Patel J, Minelli C, Gnatiuc L, Amaral AFS, Kocabaş A, et al. Prevalence and Population-Attributable Risk for Chronic Airflow Obstruction in a Large Multinational Study. Am J Respir Crit Care Med. 2021;203(11):1353-65.

6. Zein JG, Dweik RA, Comhair SA, Bleecker ER, Moore WC, Peters SP, et al. Asthma Is More Severe in Older Adults. PLoS One. 2015;10(7):e0133490.

7. Hooper R, Burney P, Vollmer WM, McBurnie MA, Gislason T, Tan WC, et al. Risk factors for COPD spirometrically defined from the lower limit of normal in the BOLD project. Eur Respir J. 2012;39(6):1343-53.

8. Mohanan S, Tapp H, McWilliams A, Dulin M. Obesity and asthma: pathophysiology and implications for diagnosis and management in primary care. Exp Biol Med (Maywood). 2014;239(11):1531-40.

9. Chowdhury NU, Guntur VP, Newcomb DC, Wechsler ME. Sex and gender in asthma. European Respiratory Review. 2021;30(162):210067.

10. Fortis S, Quibrera PM, Comellas AP, Bhatt SP, Tashkin DP, Hoffman EA, et al. Bronchodilator Responsiveness in Tobacco-Exposed People With or Without COPD. Chest. 2023;163(3):502-14.

11. Tan DJ, Lodge CJ, Walters EH, Bui DS, Pham J, Lowe AJ, et al. Can We Use Lung Function Thresholds and Respiratory Symptoms to Identify Pre-COPD? A Prospective, Population-based Cohort Study. Am J Respir Crit Care Med. 2024.

12. Lam AHS, Alhajri SA, Potts J, Harrabi I, Anand MP, Janson C, et al. Optimal spirometry thresholds for the prediction of chronic airflow obstruction: a multinational longitudinal study. ERJ Open Research. 2024:00624-2024.
